# Strain Measurements within Fibre Boards. Part II: Strain Concentrations at the Crack Tip of MDF Specimens Tested by the Wedge Splitting Method

**DOI:** 10.3390/ma5081495

**Published:** 2012-08-23

**Authors:** Gerhard Sinn, Ulrich Müller, Johannes Konnerth, Jörn Rathke

**Affiliations:** 1 Department of Material Sciences and Process Engineering, Institute of Physics and Material Sciences, BOKU–University of Natural Resources and Life Sciences, Peter Jordan Straße 82, Vienna AT-1190, Austria; 2 Wood K plus–Competence Centre for Wood Composites and Wood Chemistry, Altenberger Straße 69, Linz 4040, Austria; E-Mails: j.rathke@kplus-wood.at (J.R.); ulrich.mueller@kplus-wood.at (U.M.); 3 Department of Material Sciences and Process Engineering, Institute of Wood Technology and Renewable Resources, BOKU–University of Natural Resources and Life Sciences, Konrad Lorenzstraße 24, Tulln an der Donau 3430, Austria; E-Mail: johannes.konnerth@boku.ac.at

**Keywords:** electronic laser speckle interferometry, wedge splitting, medium density fiber board, fracture mechanics, process zone

## Abstract

This is the second part of an article series where the mechanical and fracture mechanical properties of medium density fiberboard (MDF) were studied. While the first part of the series focused on internal bond strength and density profiles, this article discusses the fracture mechanical properties of the core layer. Fracture properties were studied with a wedge splitting setup. The critical stress intensity factors as well as the specific fracture energies were determined. Critical stress intensity factors were calculated from maximum splitting force and two-dimensional isotropic finite elements simulations of the specimen geometry. Size and shape of micro crack zone were measured with electronic laser speckle interferometry. The process zone length was approx. 5 mm. The specific fracture energy was determined to be 45.2 ± 14.4 J/m^2^ and the critical stress intensity factor was 0.11 ± 0.02 MPa.

## 1. Introduction

Wood based panels consist of wood particles, fibres, flakes or veneer sheets which are usually processed with a resin and treated with pressure and heat. This procedure allows the manufacturing of products which can have dimensions and characteristics beyond those of natural wood. In the group of wood based panels, medium density fiber board (MDF) performs with the highest degree of homogeneity, due to the fibrous particle structure. The different performance of wood based panels mainly depends on the exclusion of naturally grown strength failure zones, such as for instance knots or any other type of fibre deviation. The design of such new products requires a high yield of information and a precise quality control system.

Standard testing procedures are well established in the wood based panel industry and yield valuable data for the optimization of existing products. One of these tests is the internal bond strength test [1]. The optimization of the wood based panel production process, especially the resin curing in a hot press, has to be described by analysing the core layer. The standard testing procedure of internal bond strength [1] yields only a peak value as output data, but neglects the origin of the failure. The failure onset takes place at localized flaws of the material and is therefore better described by fracture mechanics than classical strength theory.

Another standardized testing method is the measurement of bending strength according to EN 319 [2]. Bending experiments lead to pressure in the top layered face layer and tension in the bottom face layer. The specimens generally fail in the tensioned face layer and the transition zone between core layer and face layer, according to our own experiments. The bending strength describes the failure of the entire specimen, but is not able to isolate the behaviour of the core layer.

One approach, which allows a direct load transmission into the core layer of wood based panels, is the wedge splitting experiment, developed and patented by Tschegg [3]. The methodology has been successfully applied for materials like concrete [4], wood [5], laminated veneer lumber [6] and particle boards [7,8,9]. The wood panels studied were mainly loaded in plane, which yielded a failure combination of face and core layer. Instead, the core layers of MDF were tested by Matsumoto and Nairn [10] and Yoshihara [11] using DCB (Double Cantilever Beam) specimen geometry.

**Figure 1:**
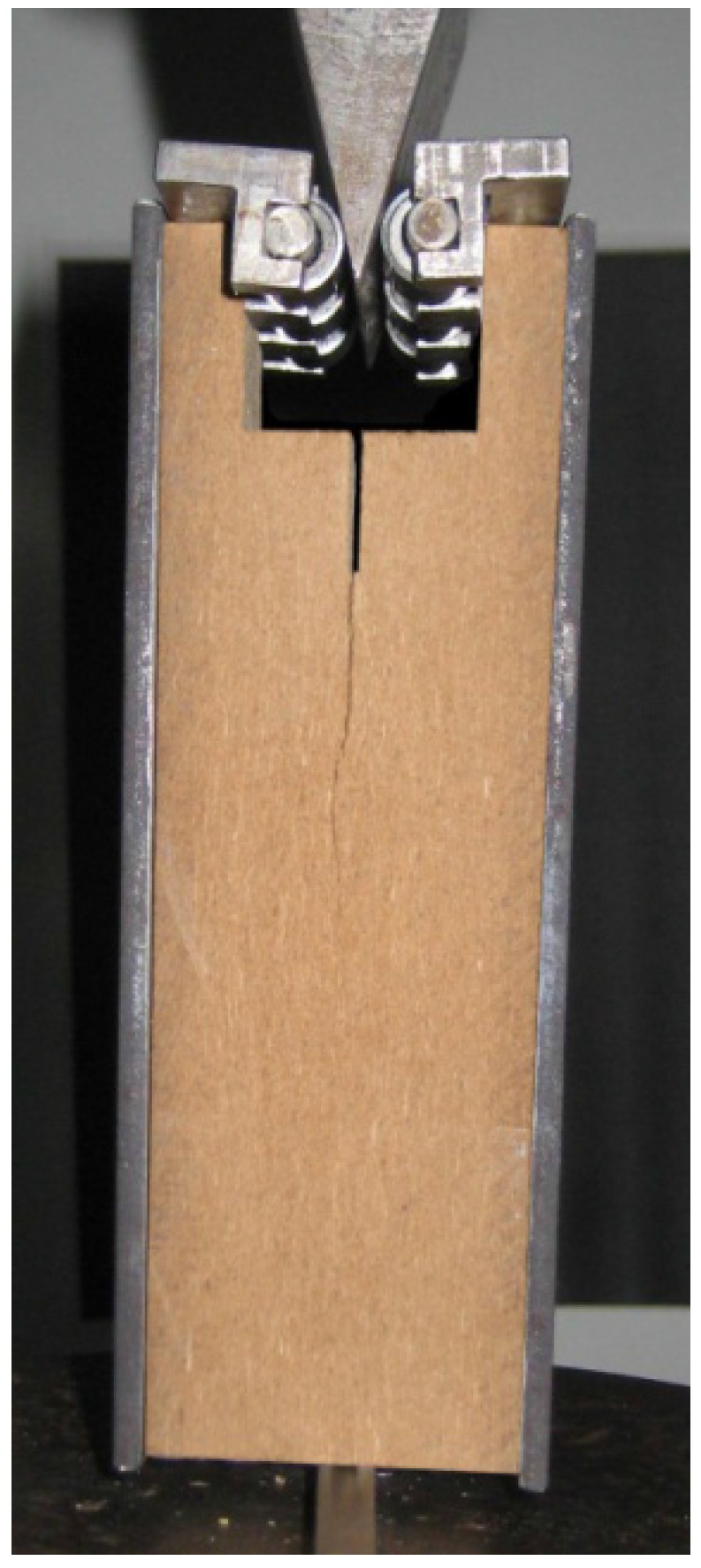
Wedge splitting test setup for the core layer testing of medium density fiber board (MDF). Specimen is reinforced by steel plates glued to the face layers of MDF.

For the analysis of the core layer of MDF, loading must be carried out perpendicularly to the plane of the board. This required some modifications to the traditional wedge splitting configuration used by Tschegg [3], where the recess for the load transmission pieces is cut into the specimen. This procedure weakens the relatively thin in-plane specimen too much; therefore, 3 mm thick steel reinforcements were glued to the MDF-specimens as reinforcement (see Figure 1). The steel reinforcements were glued to the face layer of the medium density fiberboard with a fast curing cyanoacrylate resin, yielding a stiff bonding of the steel element and the MDF. A similar modification of the wedge splitting specimens was made by Ehart *et al.* [7] for testing the core layer of particleboard: specimens were built up by three layers of the board material glued together.

An advantage of the wedge splitting experiments is that the use of the wedge increases the stiffness of the setup. The higher stiffness is favourable to the stability of the experiments [12] and an advantage over direct loading setups like CT-fracture tests or double cantilever beam experiments. According to Ehart *et al.*, [7], this test setup in combination with a precise specimen preparation and geometry allows testing under conditions of steady state crack propagation. This stable loading allows the recording of the entire load displacement curve as well as the calculation of the specific fracture energy and stress intensity factor. 

A standard value for the characterization of fracture behaviour in the field of material science is the critical stress intensity factor *K_IC_*. This is a parameter used in linear elastic fracture mechanics (LEFM). Prerequisites of LEFM are linear elastic material properties and a self-similar crack growth–properties which are only approximately satisfied for MDF since fibre bridging and micro cracking takes place [10]. Moreover, the formation of micro cracks and fibre bridging around the crack tip makes crack length measurement almost impossible [8]. 

To deal with the problems of micro cracking and fibre bridging, nonlinear elastic fracture mechanics (NLEFM) has to be applied. The concept of the total fracture energy is especially suitable. The specific fracture energy *G_f_* is described as the total fracture energy normalized by the broken area and describes an average crack resistance for the analysed material and specimen size [10]. 

Additional information on the cracking behaviour can be gained from finite element models. In this paper, finite element modelling is used to calculate the stress intensity factor. A two-dimensional, linear elastic, plane strain model of the specimen geometry was built in order to determine the critical stress intensity factor from the maximum load and the specimen stiffness. The model depends on the stiffness because the reinforcements are made of a different material (*i.e.*, steel) than the specimen.

This paper presents electronic speckle pattern interferometry (ESPI) measurements in order to determine the crack length and to verify the FE modelling. ESPI measurements were performed additionally to the wedge splitting experiments. Medium density fibre boards with a thickness of 38 mm and a mean density of 710 kg/m^3^ were used. According to Müller *et al.*, [13], ESPI measurements are a suitable tool for the validation of numerical material analysis by means of FE modelling. For a comparison with data from the literature, the stress intensity factor and the specific fracture energy were calculated. The results gained are used to analyse the appropriateness of the calculated models and to determine the applicability of the adopted wedge splitting experiment for the analysis of the core layer in wood based panels. 

## 2. Experimental Section

As described, the wedge splitting methodology has been applied to various types of materials such as concrete [4], wood [5,14,15,16,17,18,19], particleboards [7,8,9] and various others [20,21,22]. The vertical load of the universal testing machine is transferred into the specimen by a wedge of 30° and two load transmission pieces. Roller bearings are used to keep friction low. The test setup with the wedge stores less elastic energy in the machine compared to a direct loading setup and allows the transformation of vertical load into horizontal load under steady state crack development. 

The specimens were cut to dimensions of 125 mm × 24.5 mm and stored in standard climate (20°/65% RH) until the equilibrium moisture content was gained. Subsequently, a groove was sawn into the specimen’s core layer to allow the placement of the wedge and the load transmission pieces. The groove was sawn to a depth of 20 mm; then, a 10 mm long notch was sawn into the core layer for crack initiation. Ultimately, the notch was sharpened by a razor blade before testing. 

### 2.1. Specific Fracture Energy

Analyzing the load displacement curve permits characterizing the entire fracture process. The fracture energy can be calculated directly from the diagram if the crack propagation takes place under stable conditions until final fracturing of the sample occurs. The specific fracture energy *G_f_* is a material characteristic and characterizes the specimen’s resistance to crack growth. *G_f_* was calculated by dividing the integral of the load displacement curve by the fracture surface area (see Equation 1).

(1) $$G_{f}=\frac{1}{\left(L-a\right)\cdot B}\int_{0}^{L-a}Fdz$$

*B*…specimen thickness; *L*… specimen length; *a*… distance from top of the specimen to the root of the notch.

### 2.2. Stress Intensity Factor

Although it is reported that wood [5] and wood based panels [6,7] show non-linear characteristics, which is due to the large process zone and fiber bridging, nevertheless the concept of linear fracture mechanics for measuring toughness is widely accepted [5,15,23,24].

For a better understanding of the deformation and fracture process as well as to determine the critical stress intensity factor *K_IC_*, a FE simulation was performed to describe the basic mechanisms of fracture. In this calculation, the special case of the sandwich construction of the specimen, consisting of wood based panel and metal, was taken into account. The calculations were carried out with the commercial FE program ABAQUS®. The specimen was simulated as a two dimensional plane strain model. Isotropic and linear material properties were assumed. The crack tip was simulated with 36 collapsed 8-node biquadratic plane strain elements with mid-side nodes placed at ¼ of the distance along the element side to create quarter-point elements representing $1/\sqrt{r}$ stress singularity [25,26]. The ABAQUS® routine for the fracture toughness *K_I_* was used to compute the critical values. The whole model consisted of 754 elements. Several simulations with varying moduli of elasticity (Poisson ratio was fixed to 0.1) were performed in order reproduce the experimental initial slope and determine the modulus of elasticity representing the experiment. Found the modulus of elasticity, the fracture toughness was calculated by the ABAQUS® routine with the maximum load from experimental load displacement curves. 

### 2.3. Speckle Measurement

**Figure 2:**
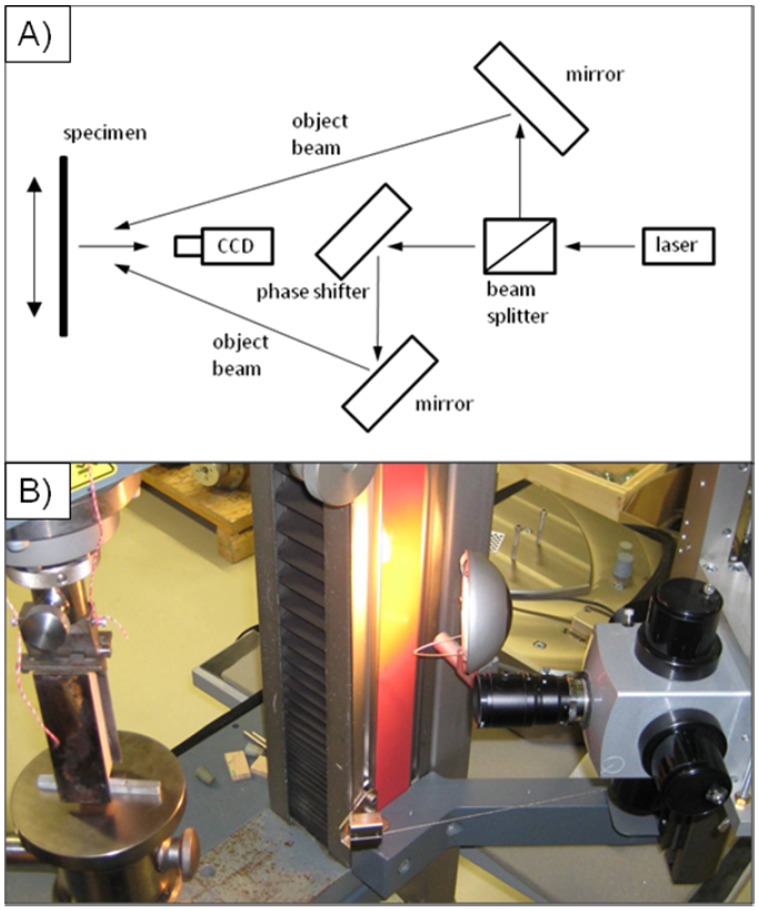
Schematic drawing of the Michelson interferometer (**a**) and electronic speckle pattern interferometry (ESPI) optics set-up for wedge splitting in-plane measurements (**b**).

The basic principle of the ESPI technique for in-plane measurements can easily be explained on the basis of the experimental setup, presented by Müller *et al.* [13]: the specimen is illuminated by two expanded laser beams. Based on the interference between the two laser beams, the laser light forms a speckle pattern which is recorded by means of a CCD camera (Figure 2a,b). Deformations on the sample surface cause a new phase difference and therefore a new speckle pattern. The calculation of changes in the image pattern is performed by subtracting the pattern from the previous image; this results in an image with typical fringe pattern [13,27]. More detailed information in terms of the ESPI technique is given by several authors such as for instance Rastogi [28], Eberhardsteiner [29], Mohan and Rastogi [30]. 

In order to analyze the stress distribution on the surface of medium density fiber board specimens with wedge splitting geometry, tests were performed on a Zwick/Roell Z020 universal testing machine. The wedge splitting specimens were placed on the bottom plate and the wedge and the load transition elements were placed in the predestined groove. A Dantec Ettemeyer Q300 (Ulm, Germany) ESPI system was mounted on the testing machine (see Figure 2b). The high sensitivity of the system requires a constant control of the Field of View while the experiment is performed. The working distance between the optical system and the specimen surface was approx. 360 mm and a total area of 44.6 mm × 38.9 mm was observed. During the experiment, the crosshead was moved with a speed of 0.1 mm/min. Pictures were taken after three fringes, which yielded approx. 15 pictures per specimen.

The analysis of the total sample deformation was performed as described in Part 1 of the trilogy [31].

## 3. Results and Discussion

In this paper, the wedge splitting experiment [3] was applied to specimens of medium density fiber boards (MDF) with a thickness of 38 mm and an average density of 710 kg/m^3^. The specific fracture energy was calculated from the load displacement curves and the stress intensity factors were calculated using a FE simulation. To validate the FE simulation and to analyze crack length, ESPI measurements were performed.

### 3.1. Specific Fracture Energy

Measurements of the fracture energy *G_f_* were performed with nine specimens and yielded a mean value of 45.2 J/m^2^ ± 14.4 J/m^2^. Matsumoto and Nairn [10] found values of 48.4 J/m^2^ for the initiation toughness *G_C_* of MDF for the same loading direction but a mean density of 737 kg/m^3^ and 48.2 J/m^2^ for specimens with a density of 609 kg/m^3^. Both kinds of materials had the same thickness of 19 mm. Using the values from the crack resistance curve (R-curve) provided by Matsumoto and Nairn [10], the specific fracture energy can be compared to the wedge splitting experiments with (L − a) = 90 mm as follows:

(2) $$G_{f}=\frac{1}{\left(L-a\right)}\int_{0}^{U_{\text{max}}}dU=\frac{1}{\left(L-a\right)}\int_{0}^{L-a}Rda=\frac{1}{\left(L-a\right)}\int_{0}^{L-a}\left(G_{C}+Slope\;a\right)da=G_{C}+\frac{1}{2}Slope\left(L-a\right)$$

The R-curve was approximated by a linear equation by Matsumoto and Nairn [10]. The results and the specific fracture energy derived from Matsumoto and Nairn [10] are summarized in Table 1. Results for *G_f_* extrapolated from the data of Matsumoto and Nairn are approx. 30% higher than current experimental results. 

**Table 1:** Initiation toughness G_C_, slope of rising R-curve from Matsumoto and Nairn (1) [10] and specific fracture energy G_f_ predicted according to Equation 2 for a ligament length of 90 mm (2).

Panel	G_C_^1^ (J/m^2^)	Slope^1^ (J/m^3^)	G_f_^2^ (J/m^2^)
609 kg/m^3^ (19 mm)	48,2	296	61,52
737 kg/m^3^ (19 mm)	48,4	303	62,04

### 3.2. Stress Intensity Factor

The stress intensity factors were calculated using the described finite element simulation. The mean value for the critical stress intensity factor for the tested medium density fiberboard was *K_IC_* = 0.111 ± 0.015 Mpam^0.5^. Only a few experiments analyzing the fracture toughness of MDF were found in the literature. Niemz *et al.*, [23,24] used CT specimens according to ASTM 399 to analyze the stress intensity factor. The specimens were oriented parallel to the board plane and yielded *K_IC_* values of 1.81 ± 0.33 MPam^0.5^ (CV 18.2%) for a density of 710 kg/m^3^ (20 °C/65% RH) and numbers in a range of 0.36 ± 0.03 MPam^0.5^ (8.3% CV) to 1.29 ± 0.06 MPam^0.5^ (CV 4.7%) with a density of 500 kg/m^3^, depending on the equilibrium moisture content, which varied from 21.4% to 3.5%. The differences between our data and the data from Niemz *et al.*, [23,24] can be traced back to the fact that the specimens were tested perpendicularly to experiments presented here and that these literature values reflect a combination of face layer and core layer. Matsumoto and Nairn [32] used modified CT specimens for testing the middle layer of MDF. They provided experimental results for the initiation toughness *G_c_* and the modulus of elasticity *E* from simulations of 19 mm thick MDF boards. To compare their results with the results presented here, the well-known equation from linear elastic fracture mechanics was used:

(3) $$K_{Ic}=\sqrt{G_{Ic}\frac{E}{1-\upsilon^{2}}}$$

The results for MDF 46, given in Table 2, are in good agreement with our own results of *K_IC_* = 0.111 ± 0.015 Mpam^0.5^. The labeling “MDF 38” and “MDF 46” reflects the density of the specimens in 38 lbs/ft^3^ and 46 lbs/ft^3^. 

**Table 2:** Initiation toughness, modulus of elasticity and Poisson’s ratio from [32]; K_IC_ calculated according to Equation 2.

	Density, (kg/m^3^)	G_c_, (J/m^2^)	E, (MPa)	ν, (-)	K_IC_, (MPam^0.5^)
MDF 38	609	59	90	0.33	0.077
MDF 46	737	48	200	0.33	0.104

### 3.3. ESPI Measurement

**Figure 3:**
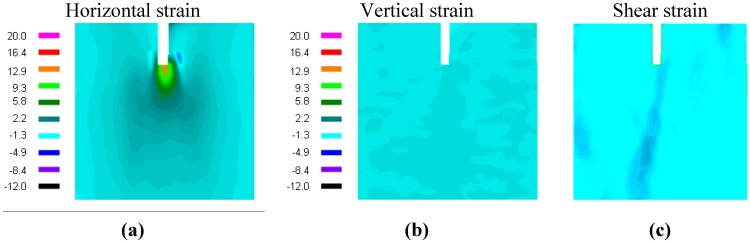
(**a**) measured in-plane horizontal strain distribution ε_xx_ (µm/mm); (**b**) measured vertical strain profile ε_yy_; and (**c**) shear strain profile.

In Figure 3 the ESPI strain results for one representative specimen are shown. The predominantly mode I horizontal strain is visible in the left image of Figure 3; the vertical and the shear strain are of minor magnitude.

**Figure 4:**
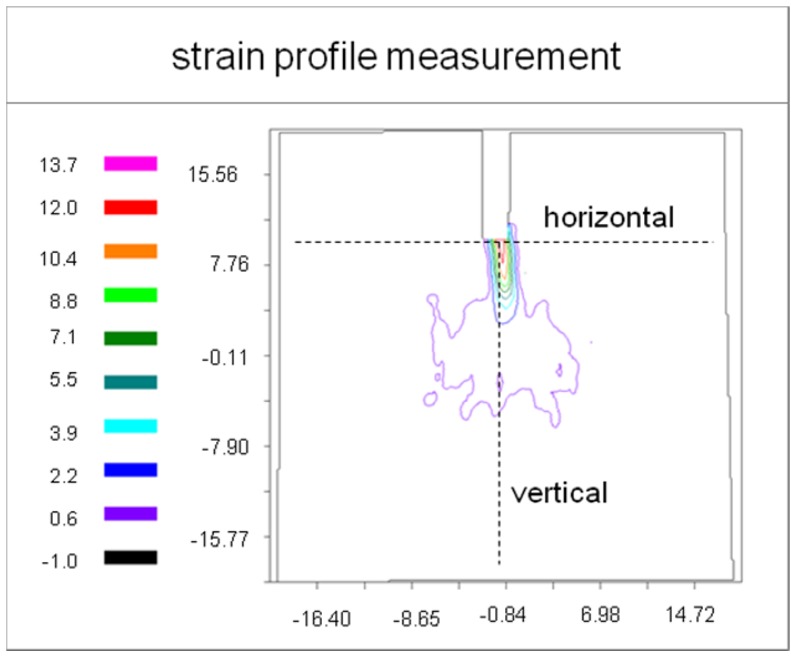
Contour graph of *ε_xx_* (µm/mm) showing the intersection lines were the horizontal and vertical profiles were extracted.

**Figure 5:**
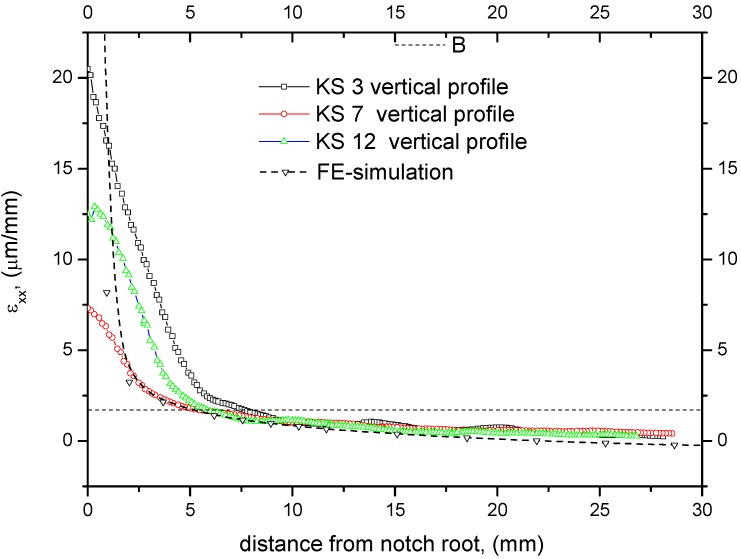
Vertical profile lines of e_xx_ in crack growth direction from ESPI measurements and from simulation. Dashed horizontal line shows critical strain at failure onset. Fracture process zone length can be determined within 5 to 10 mm.

**Figure 6:**
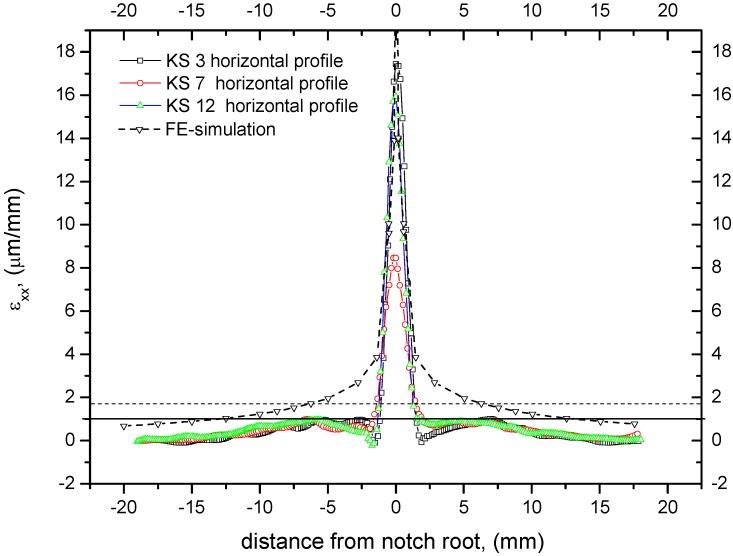
Horizontal profile lines of e_xx_ experimental and simulated. Dashed line represent strain at yielding providing a process zone with of 2.4 mm (acc. to method 1), whereas solid horizontal line touches the first maxima at left and right side of the center peak leading to a process zone width of 6.3 mm (acc. to method 2).

For a quantitative analysis profile lines according to Figure 4 were extracted from the ESPI profiles. Horizontal strains *ε_xx_* were examined along the horizontal and vertical lines shown in Figure 4. Profile lines shown in Figure 5 and Figure 6 were used to determine the size of the fracture process zone.

Using the strain profiles, the deformation zone size was measured. Three different methods were applied: First, we used the average values of the load and the initial slope as input data for the FE simulation to determine an average isotropic modulus of elasticity (*E* = 298 MPa). This approximated modulus of elasticity was used in combination with the internal bond strength *σ*_ib_ = 0.51 ± 0.19 MPa [31] to calculate the critical strain at failure. The calculation of corresponding strain yielded

(4) $$\varepsilon =\frac{\sigma }{E}=\frac{0.51}{298}=1.71\frac{\mu \text{m}}{\text{mm}}$$

The width the process zone determined using Method 1 provided only half of the value achieved for the vertical profile (see Figure 6). A closer look at the horizontal profile lines shows that approaching the center of the specimen, the profile runs through local maxima. These maxima might be caused by the initiation of micro-crack-formation beyond this point and therefore be classified as the border of the process zone (Method 2). This assumption can be justified by the solution of the theoretical stress field surrounding a crack tip, which is proportional to the inverse of the square root of the distance from the crack tip in the linear elastic region. Within the micro cracking region the mathematical relation is different. The border can be allocated to the maxima described before. Results are, once again, are summarized in Table 3, column 4.

**Table 3:** Electronic speckle pattern interferometry (ESPI) results of process zone size r_p_.

Specimen	Process zone length in crack forward direction from vertical profiles in (mm); Method 1	Process zone width in (mm) from horizontal profiles; Method 1	Process zone width in (mm) from horizontal profiles; Method 2
1 (KS 3)	7.66	2.44	6.23
2 (KS 7)	5.47	2.75	6.58
3 (KS 12)	5.83	2.57	6.12

Intersection of the horizontal dashed line in Figure 5, corresponding to the critical strain, with the profile lines gives the process zone size (Method 1). This method worked well for the vertical profile shown in Figure 3 and Figure 4. The results are summarized in Table 3.

The third method to estimate the fracture process zone size in crack growing direction uses the analytical solution of the stress field surrounding a crack [33]. Equation 5 provides the second order estimate of process zone length for plane strain conditions (see Equation 2.68 for *r_y_* in [33]). 

(5) $$r_{p}=2r_{y}=\frac{1}{3\pi }{\left(\frac{K_{I}}{\sigma_{ib}}\right)}^{2}=\frac{1}{3\pi }{\left(\frac{0.111}{0.51}\right)}^{2}=5.03\;\text{mm}$$

Equation 5 is an approximation since it was derived for an ideal elastic plastic material [33]. The micromechanics of damage of MDF are different from this assumption. Exceeding a critical load micro cracks develop in the material and prevent the stress from increasinge further in the concerned region. Although the micromechanics of cracking is different from that of ideal material, a process zone develops around the crack tip similar to a plastic zone in metals. The analytically determined process zone width r_pw_ is approximately 1.25 times the process zone in forward direction [33]; for a Poisson ratio of 0.1, it gives r_pw_ = 6.3 mm. This value is close to the second method of experimental evaluation. It might be concluded from the process zone size and shape, comparing the ESPI results with the FE simulation (see Figure 5 and Figure 6), that the material behaves isotropically within the range of measurement. This conclusion is supported by the well-known correlation between the modulus of elasticity and the density. The density profile shown in part one of this series [31] is approx. constant within ±10 mm from center; therefore the isotropic FE-simulation might describe the material and setup correctly within the measurement plane and region. Nevertheless, there might be a different modulus of elasticity in the direction of depth.

## 4. Conclusions

Wedge splitting experiments were performed in order to characterize the fracture behavior of the MDF core zone. Compared to the original, patented setup, specimens with metal reinforcements at the sides were used. This modification was necessary to guarantee that the highest stresses within the specimen occur at notch ground and avoid failing of the specimen close to the surface. 

ESPI measurements were used to measure the process zone and visualize the material inhomogeneity. The ESPI results were compared to linear elastic and isotropic FE simulations and confirm the assumptions of isotropic material behavior made for the FE simulations.

The article presents new experimental data on fracture toughness of the MDF core layer described in terms of specific fracture energy and critical stress intensity factor. Results show that fracture experiments performed can provide valuable information in addition to the standardized tests and characterize the core material of MDF. The modified wedge splitting setup in combination with two-dimensional strain measurement and FE-simulations can provide further information on nonlinearities or anisotropic material response in an early state of damage.
